# Inflammatory-driven NK cell maturation and its impact on pathology

**DOI:** 10.3389/fimmu.2022.1061959

**Published:** 2022-12-09

**Authors:** Elsa Bourayou, Rachel Golub

**Affiliations:** Institut Pasteur, Université Paris Cité, INSERM U1223, Lymphocyte and Immunity Unit, Paris, France

**Keywords:** NK cells, bone marrow, development, maturation, inflammation

## Abstract

NK cells are innate lymphocytes involved in a large variety of contexts and are crucial in the immunity to intracellular pathogens as well as cancer due to their ability to kill infected or malignant cells. Thus, they harbor a strong potential for clinical and therapeutic use. NK cells do not require antigen exposure to get activated; their functional response is rather based on a balance between inhibitory/activating signals and on the diversity of germline-encoded receptors they express. In order to reach optimal functional status, NK cells go through a step-wise development in the bone marrow before their egress, and dissemination into peripheral organs *via* the circulation. In this review, we summarize bone marrow NK cell developmental stages and list key factors involved in their differentiation before presenting newly discovered and emerging factors that regulate NK cell central and peripheral maturation. Lastly, we focus on the impact inflammatory contexts themselves can have on NK cell development and functional maturation.

## Introduction

NK cells are part of the innate lymphoid cell (ILC) family and are involved in a large variety of contexts. They are crucial in the immunity to intracellular pathogens as well as cancer due to their ability to kill infected or malignant cells (1). In contrast to T cells, NK cells do not require antigen exposure to get activated and start producing pro-inflammatory cytokines and/or mediating cytolytic activities. Their functional response is rather based on a balance between inhibitory/activating signals and on the diversity of germline-encoded receptors expressed at their surface. NK cells share a lot of similarities with type 1 ILCs (ILC1s), formerly termed “tissue-resident NK cells” (2, 3). They both secrete type 1 cytokines such as tumor necrosis factor alpha (TNFα) and interferon gamma (IFNγ) and can be activated by interleukin (IL)-12, IL-15 and/or IL-18. However, to the exception of few tissues such as liver, most ILC1s are non-cytotoxic cells (4).

In order to reach optimal functional status, NK cells go through a step-wise development in the bone marrow before their egress. After, dissemination into peripheral organs *via* the circulation (5), they can undergo their final stages of maturation. Because NK cells harbor a strong potential for clinical and therapeutic use, it is important to continue unveiling the molecular mechanisms regulating their development and maturation especially in inflammatory contexts. In this review, we first summarize bone marrow NK cell developmental stages and list key factors involved in their differentiation such as γc-chain related cytokines and transcription factors. Then, we present newly discovered and emerging factors regulating NK cell maturation and functional response which could constitute new targets for NK cell-base therapies. Lastly, we discuss the possible impact of the inflammatory context on NK cell maturation and the detrimental or beneficial consequences on the physiopathology.

## Development and functions of NK cells

### Murine and human developmental stages in the bone marrow

**Figure 1:**
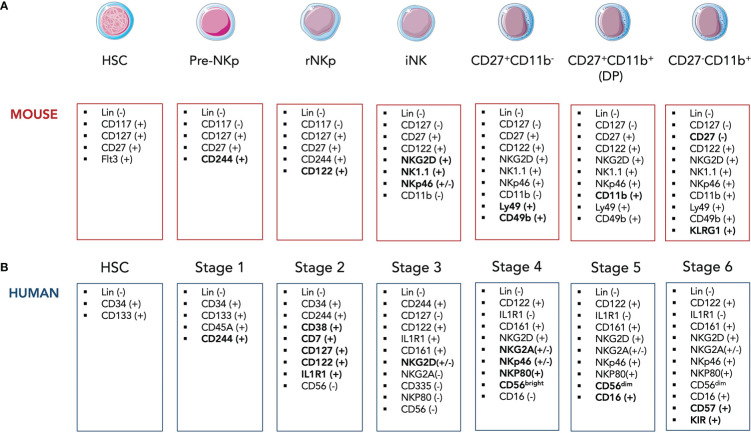
Simplified model of developmental stages of murine **(A)** and human **(B)** NK cells in the bone marrow. HSC, hematopoietic stem cell; CLP, common lymphoid progenitor; rNKp, refined NK progenitor; iNK, imature NK cell.

In adult mice, the hematopoietic stem cells (HSCs), precursors of all leukocytes and erythrocytes, can differentiate into common lymphoid progenitor (CLP) cells defined as Lin^-^ cKit^low^ IL7Rα (CD127)^+^ Flt3^+^ (6). The CLP cells give rise to pre-T, pro-B cells, common innate lymphoid progenitor (CILP) cells as well as pre-NK progenitors (pre-NKp) expressing CD244 (2B4) which then differentiate into refined NK progenitors (rNKp) (7, 8). The commitment to the NK lineage is marked by the expression of the IL-2 receptor β chain (CD122) (**Figure 1A**) (9). The rNKps then progress toward the immature NK (iNK) stage which is itself divided into several stages, each characterized by the appearance of lineage-specific markers. Thus, iNK cells acquire in a sequential manner C-type lectin-like activating receptors NKG2D and CD161 (NK1.1), and the natural cytotoxicity receptor 1 (NCR1) also termed NKp46 (**Figure 1A**) **(** 10, 11). iNK cells are capable to secrete IFNγ and were shown to mediate TRAIL-dependent cytotoxicity (12, 13). Functionally NK cells arise from iNK cells and require the acquisition of CD49b and Ly49 receptors that recognize MHC-I molecules (14). The functional maturation of NK cells is usually divided into 3 stages based on different expression levels of CD27 and CD11b markers: CD27^+^ CD11b^-^ cells give rise to double positive (DP, **Figure 1A**) cells which then differentiate into the final CD27^-^ CD11b^+^ stage (14). Immature CD27^+^ NK cells display the proeminent proliferative potential. The two CD11b^−^ CD27^+^ and CD11b^+^ CD27^+^ NK cell stages present the best ability to secrete cytokines with CD27 being directly implicated in this process (15, 16). Therefore, the terminal stage of maturation is marked by the expressions of CD11b (Mac-1), CD43 and KLRG1 as well as the downregulation of the CD27 marker (**Figure 1A**) (15, 17). Terminally differentiated CD27^-^ CD11b^+^ mature (m)NK cells have increased cytotoxic activity. Hence, along they terminal maturation, NK cells slowly increase their cytotoxic capacity while reduce their possibilities for homeostatic expansion (15).

Although ILC1s and NK cells share a common lymphoid progenitor, they mostly arise from distinct developmental pathways (18, 19). Nonetheless, Id2^+^ progenitors were shown to be able to give rise to both helper ILCs and NK cells (20). Early lymphoid progenitors with innate specificities were identified in the BM as TCF-1^+^ cells (21). Mechanisms that drive helper versus cytotoxic lineages are still unclear as only a minor fraction of NK cells are labelled by Zbtb16 suggesting a main NK cell developmental pathway upstream of the common helper innate lymphoid progenitor (CHILP) (18, 22). Globally, ID2^+^ CHILP can give rise to all ILC subsets while the more committed PLZF^+^ precursor is giving rise to mature ILC1s, ILC2s or ILC3s (18, 20, 22). Recently, ILC1s present in the peripheral tissues were described as TCF-1^hi^ precursors that could differentiate *in situ* into cytotoxic-like ILC1 through the upregulation of the Hobit transcription factor (23). Moreover, some hematopoietic progenitors Lin^-^Sca1^+^Mac1^+^, called LSM were detected in the adult liver and were able to differentiate into diverse hematopoietic fates despite a preference for ILC1s over NK cells (24).

In humans, NK cell development is composed of 6 stages which are quite similar to those in mice although several surface markers differ between the two species. Lin^-^ CD34^+^ CD133^+^ human HSCs differentiate into Lin^-^ CD34^+^ CD244^+^ pre-NKp cells (Stage 1) that irreversibly commit to NK lineage through the expression of CD122 as well as the IL-1β receptor IL1R1 (**Figure 1B**) (25). Human rNKPs (stage 2) then give rise to human equivalent of iNK cells (Stage 3) through the expression of CD161 and NKG2D (26). The appearance of CD56 marks the transition from Stage 3 to Stage 4 along with NKp46, NKp30, NKp80 and NKG2A expressions (**Figure 1B**) (27). NK cells can be further divided based on their surface expression level of CD56. CD56^bright^ NK cells are considered more immature (Stage 4) and are able give rise to CD56^dim^ NK cells which start upregulating CD16 (FcγRIII) at the same time (Stage 5). Most terminal mature NK stage (Stage 6) is defined by the expression of CD57 and killer cell immunoglobulin-like receptors (KIR), the latter representing the human counterparts of mice Ly49 receptors (**Figure 1B**) (28). While CD56^bright^ NK cells are considered strong producers of pro-inflammatory cytokines, CD56^dim^ NK cells harbor an enhanced cytotoxicity. However, Wagner et al. challenged this paradigm by demonstrating that IL-15 priming of CD56^brigh^ NK cells resulted in an enhanced and potent antitumor response of these cells *in vitro* and in *in vivo* murine xenograft models (29). Interestingly, an intermediate subset of CD56^dim^ NK cells expressing CD62L was identified (30). CD56^dim^ CD62L^+^ NK cells displayed high proliferative ability as well as IFNγ secretion after cytokine stimulation in a similar manner as CD56^bright^ NK cells. Yet, CD62L^+^ and CD62L^-^ CD56^dim^ but not CD56^bright^ NK cells were able to respond to activating receptor stimulation.

NK cells primarily develop in the bone marrow (BM) although some studies suggested the existence of peripheral and thymic development of NK cells. Briefly, GATA-3^+^ CD127^+^ NK cells have been identified in the murine thymus with a distinct origin from BM NK cells (31). These thymus-derived NK cells were able to repopulate peripheral lymphoid organs and presented a phenotype similar to that of CD56^bright^ NK cells in humans with decreased cytotoxicity but potent cytokine production. NK cell development can also take place in periphery (liver, spleen, lymph nodes) although this peripheral maturation likely results from the migration of BM NK progenitors rather than distinct developmental pathways (32).

### Molecules driving NK cell development and maturation

#### γc chain cytokines

Many cytokines are involved in NK cell development and maturation. Among them is the family of common gamma (γc) cytokines composed of several interleukins (IL-2, IL-4, IL-7, IL-9, IL-15 and IL-21) all signaling through the transmembrane glycoprotein γc chain (33). Its importance in NK cell development is highlighted by the fact that γc^-/-^ mice lack NK cells as well as all helper ILCs (34).

While IL-7 is necessary for lymphoid development with CLPs expressing high levels of IL-7Rα, mice deficient in IL-7 or IL-7Rα actually display normal NK cell maturation in the BM indicating that IL-7 is not required for NK cell development (35, 36). Human IL-7 knocked-in in NOD Scid Gamma (NSG) mice also revealed that IL-7 is not sufficient to induce human NK cell development (37). However, it was shown that murine thymic CD127^+^ NK cell homeostasis depends on IL-7 (31). In humans, Michaud et al. demonstrated that IL-7 enhanced the survival of CD56^bright^ NK cells by increasing BCL2 expression although their cytoxicity, cytokine production and activation marker expression were not impacted (38).

IL-2 and IL-15 both signal through the γc and IL-2Rβ chains but differ in their alpha subunit. They respectively trigger STAT1 and STAT5. IL-2Rα (CD25) is upregulated on activated NK cells and drives a IL-2-dependent proliferation and production of the cytotoxic molecules perforin and granzyme B. IL-2 can also play a role in human NK cell survival (39). Yet, IL-2-deficient mice exhibit normal NK cell development (40).

On the other hand, mice lacking IL-15, IL-15Rα or IL-2Rβ display specific NK cell near-loss revealing an essential and non-redundant role for this cytokine in NK cell development and maturation (41–43). Transgenic overexpression of IL-15 leads to increased NK production (44). Several studies demonstrated that IL-15 also regulates human NK cell homeostasis. Using Rag2^−/−^γc^−/−^ mice transplanted with human hematopoietic stem cells, Huntington and al showed that human IL-15 induced NK cell proliferation and differentiation leading to an accumulation of CD16^+^KIR^+^ mature NK cells (45). An *Il2RB* mutation in siblings leading to the dysregulation of IL-2/IL-15 signaling revealed an expansion in CD56^bright^ NK cells but a strong defect in terminally mature NK cells causing ultimately autoimmunity and increased susceptibility to Cytomegalovirus (CMV) infection (46).

IL-15 acts on cells as soon as CD122 is upregulated at the rNKP stage and continues to drive their maturation into mNK cells. It can signal through IL-2Rβ/γc heterodimer either alone with poor affinity or through transpresentation *via* IL-15Rα with high affinity. The latter consists in IL-15 binding to IL-15Rα expressed at the surface of presenting cells (dendritic cells, macrophages, stromal cells…) and being transpresented to IL-2Rβ/γc-expressing NK cells (47–49). The importance of this mechanism was demonstrated with adoptive transfer experiments where the injected NK cell pool was not maintained in IL-15Rα-deficient hosts while IL-15Rα-deficient NK cells transferred in wild-type (WT) mice were able to survive (50). IL-15 is also involved in murine and human NK cell survival and proliferation both in central and periphery (51, 52).

IL-21 was shown to synergize with IL-2 resulting in an increase of NKG2A, CD25, CD86, CD69, perforin and granzyme B expression in human CD56^bright^ NK cells (53). IL-21 was sufficient to maintain human NK cells alive in contrast to their murine counterpart to which IL-21 alone fails to sustain their survival. A cell line with membrane bound IL-21 was shown to induce robust and sustained proliferation of human NK cells (54). The expanded NK cells displayed higher cytotoxic activity against blood and solid tumor cells when compared to IL-2-activated NK cells. Mechanistically, membrane-bound IL-21 led to the activation of STAT3/c-Myc pathway and promoted aerobic glycolysis in NK cells. This “NK cell feeder cell line” constitutes a novel strategy enabling expansion and activation of NK cells prior to their use in clinical studies and therapies.

Altogether, this places NK cells at the center of multiple interactions with γc chain cytokine-producing cells.

#### Transcription factors

NK cell development is regulated by a vast network of transcription factors acting sequentially from the earliest progenitor to the terminally differentiated stage. We selected only a few of them, some playing a role during early development and others during late maturation.

Nfil3 (also termed E4BP4) specifically impact NK cell development as Nfil3-deficient mice display a specific lack of NK progenitor cells and mature NKs despite an early expression among CLPs (55, 56). Interestingly, it was demonstrated that deletion of Nfil3 at the rNKP stage (or later stages) did not influence NK cell numbers nor their functional response (57). On the other hand, ectopic expression of Nfil3 in CLPs was able to restore NK cell development in Nfil3^-/-^ mice (58). Altogether, this suggests that Nfil3 is required for NK cell lineage commitment, particularly at the transition from CLP to rNKP but is dispensable for NK cell maturation.

Another key transcription factor in NK cell development is STAT5, downstream of IL-15. When IL-15 (or IL-15/IL-15Rα complex) is presented to the cell, the Janus Kinase (JAK)-1 and JAK-3 are respectively recruited to IL-2Rβ and γc leading to STAT3 and STAT5 phosphorylation. The importance of the JAK-1/3-STAT5 pathway in maintaining NK cell development has been known for decades with JAK-3- and STAT5b-deficient mice displaying severe lack of NK cell maturation and function (59, 60). Conditional deletion of STAT5 in NCR1-positive cells also showed an abrogation of NK cell maturation in the BM (61) confirming the crucial role of STAT5 in NK cell development.

The highly homologous T-box transcription factors T-bet and Eomesodermin (Eomes) are known to be essential and necessary for NK cell development and maturation. In *Tbx21* ^-/-^ mice, NK cell numbers are significantly lower in periphery however slightly higher in the BM (62). It was shown that T-bet can bind to the S1pr5 locus and thus promotes its expression (63). Decreased expression of S1pr5 in *Tbx21* ^-/-^ mice then leads to an impaired NK cell egress from the BM which can explain the drop in peripheral NK cells (64). T-bet was also shown to promote NK cell maturation as T-bet-deficient NK cells display lower levels of several maturation markers such as CD11b, DX5 and CD43 (62). Conditional deletion of Eomes in NCR1-positive cells leads to a compromised NK cell development with decreased NK cells in the periphery as well as in the BM (65, 66). Acquisition of DX5, Ly49 receptors and cytotoxicity were shown to be dependent upon Eomes and deletion of Eomes in NK cells led to a reversion to a more immature stage (67). Finally, loss of both transcription factors resulted in a complete depletion of NK cells (67) supporting the idea that T-bet and Eomes, while acting at different stages of maturation, also play redundant roles and can partially compensate for one another.

Downstream of T-bet is the transcriptional repressor B lymphocyte-induced maturation protein 1 (Blimp-1) encoded by *Prdm1* (68). Blimp-1 is induced by IL-15 and controls cytokine production in both human and murine NK cells (68, 69). Blimp-1 is required for NK cell homeostasis and maturation and negatively regulates their proliferation. Loss of Blimp-1 results in decreased expression of maturation markers KLRG1 and CD43.

#### mTOR pathway

Another key regulator of NK cell development is the mechanistic target of Rapamycin (mTOR) pathway. mTOR is a highly conserved serine/threonine kinase capable of integrating two structurally distinct complexes, mTOR complex 1 (mTORC1) and mTORC2. mTORC1 is composed of mTOR, Raptor, DEPTOR, PRAS40 and mLST8 proteins while mTORC2 comprises mTOR, Rictor, DEPTOR, mLST8, Proctor1/2 and mSin1 (70). mTORC1 is sensitive to its inhibitor rapamycin whereas mTORC2 is mostly insensitive to it. These two complexes regulate various biological processes such as proliferation, cell growth and metabolism. Downstream of the IL-15 receptor IL-2Rβ/γc is the PI (3)K-mTOR pathway which can initiate transcription related to NK cell maturation and function. PI (3)K can activate mTORC1 through the canonical PI3K-PDK1-Akt-TSC1/2-mTORC1 pathway as well as mTORC2 although less is known about the precise mechanism (70). Given that IL-15 can directly initiate mTORC1 and mTORC2 activation, it is tempting to speculate that mTOR signaling pathway is involved in the regulation of NK cell development and function. Indeed, Yang et al. showed that in mTORC1-deficient *Ncr1^Cre^ x Raptor^flox^* mice, NK cell maturation was blocked at the CD27^+^ CD11b^-^ NK stage in the BM while mTORC2-deficient *Ncr1^Cre^ x Rictor^flox^* mice displayed an arrest at the DP NK stage (71). These results suggest that both complexes are required for NK cell maturation yet in a non-redundant manner. Mechanistically, mTORC1 induces Eomes expression while mTORC2 inhibits Forkhead transcriptions factor of the O class-1 (FOXO1), a gene highly expressed in iNK and CD27^+^ CD11b^-^ NK cells (71). FOXO1 is a negative regulator of NK cell development (72) thus by suppressing FOXO1 expression, mTORC2 promotes terminal differentiation of NK cells.

Interestingly, mTOR is a target of TGF-β, and the latter was shown to inhibit the functional activation of NK cells by repressing the mTOR pathway (73). *In vitro* treatment of murine and human NK cells with recombinant TGF-β abrogated IL-15-mediated activation of mTOR. *In vivo* constitutive TGF-β signaling and mTOR depletion both led to the arrest of NK cell development. Deletion of TGFβRII enhanced IL-15-driven mTOR activation and NK cell cytotoxicity. Activin A, a member of the TGF-β superfamily was also shown to induce phosphorylation of SMAD2/3 in mouse and human NK cells leading to the impairment of IL-15-mediated NK cell proliferation and granzyme B production (74). However, suppression of TGF-β signaling did not impact NK cell development (73). In 2018, Zaiatz-Bittencourt et al. also demonstrated that canonical TGF-β signaling pathway does repress human NK cell metabolism and functions (75). Yet, in their study, the repression of NK cell metabolic responses did not involve the inhibition of mTORC1 (75) in contrast to the results from Viel et al. (73).

### NK cell effector functions

NK cells are part of the first line in the host defense against intracellular pathogens and tumor cells. Their functional response is dependent on the integration of signals through either inhibitory or activating germline-encoded receptors. The Ly49 receptors in mice and their human equivalent killer cell immunoglobulin-like receptors (KIR) represent the major family of NK cell inhibitory receptors and recognize the MHC class I molecules expressed by healthy cells (76, 77). This protects the host against auto-reactivity. However, tumor cells or pathogen-infected cells lose MHC I molecules expression leading to less inhibition of NK cells. At the same time, NK cells are activated through stimulation of their activating receptors such as natural-killer group 2 member D (NKG2D) through binding to target cell-derived ligands (78).

Once activated and functional, NK cells can act *via* two distinct mechanisms: cytotoxic immune response and secretion of cytokines. The NK cell cytotoxic response itself comprises two processes. One of them consists in a direct action through the release of cytolytic granules. An immunological synapse (IS) is formed between the NK cell and its target cell followed by a reorganization of the cytoskeleton (79). The microtubule organizing center and the secretory lysosomes are polarized towards the IS (80). The secretory lysosomes then fuse with the plasma membrane of the target cell and the cytolytic molecules are released in the cytoplasm. There, the perforin polymerizes and forms pores at the membrane allowing the serine protease granzymes to enter (81). Granzymes trigger apoptosis in a caspase-dependent manner (82) or through proteolytic cleavage of pro-apoptotic protein Bid (83). The second process by which NK cell can kill target cells is indirect and mediated by the engagement of death receptors present at the surface of target cells such as TNF-related apoptosis-inducing ligand-receptor (TRAIL-R) and Fas (CD95). NK cells express the corresponding ligands (TRAIL and FasL) which trigger caspase 3-dependent apoptosis of the target upon binding (84, 85).

Another mechanism in NK cell functional response is the secretion of cytokines and other inflammatory factors. Although activated NK cells can produce a vast variety of pro-inflammatory or immunosuppressive molecules, they primarily secrete Th1-type cytokines such as IFNγ, TNFα and granulocyte/monocyte colony-stimulating factor (GM-CSF). They can also produce chemokines such as CCL3 (MIP-1α), CCL4 (MIP-1β), CCL5 (RANTES), XCL1 (lympho-toxin), and CXCL8 (IL-8) (86, 87). The secretion of these factors directly contributes to the activation and recruitment of other immune cells (T cells, DCs, monocytes and macrophages…) to the site of inflammation (87). These same cells can also influence the NK cell cytokine production by secreting activating cytokines. For instance, DCs can produce IL-12, IL-15 and IL-18 all three being able to activate NK cells (88). IL-12 significantly enhances their production of IFNγ in a STAT4-dependent manner while IL-18 signaling promotes *Ifng* transcript expression through induction of AP-1 and p38 MAPK (89, 90).

## Factors modulating NK cell maturation and functional response

NK cell terminal maturation is the key to obtaining differentiated effector cells with optimal antiviral and antitumor properties. The identification of factors modulating NK cell maturation and consequently their functional response has been at the center of many studies over the past few years.

### Central regulation of NK cell development

**Figure 2:**
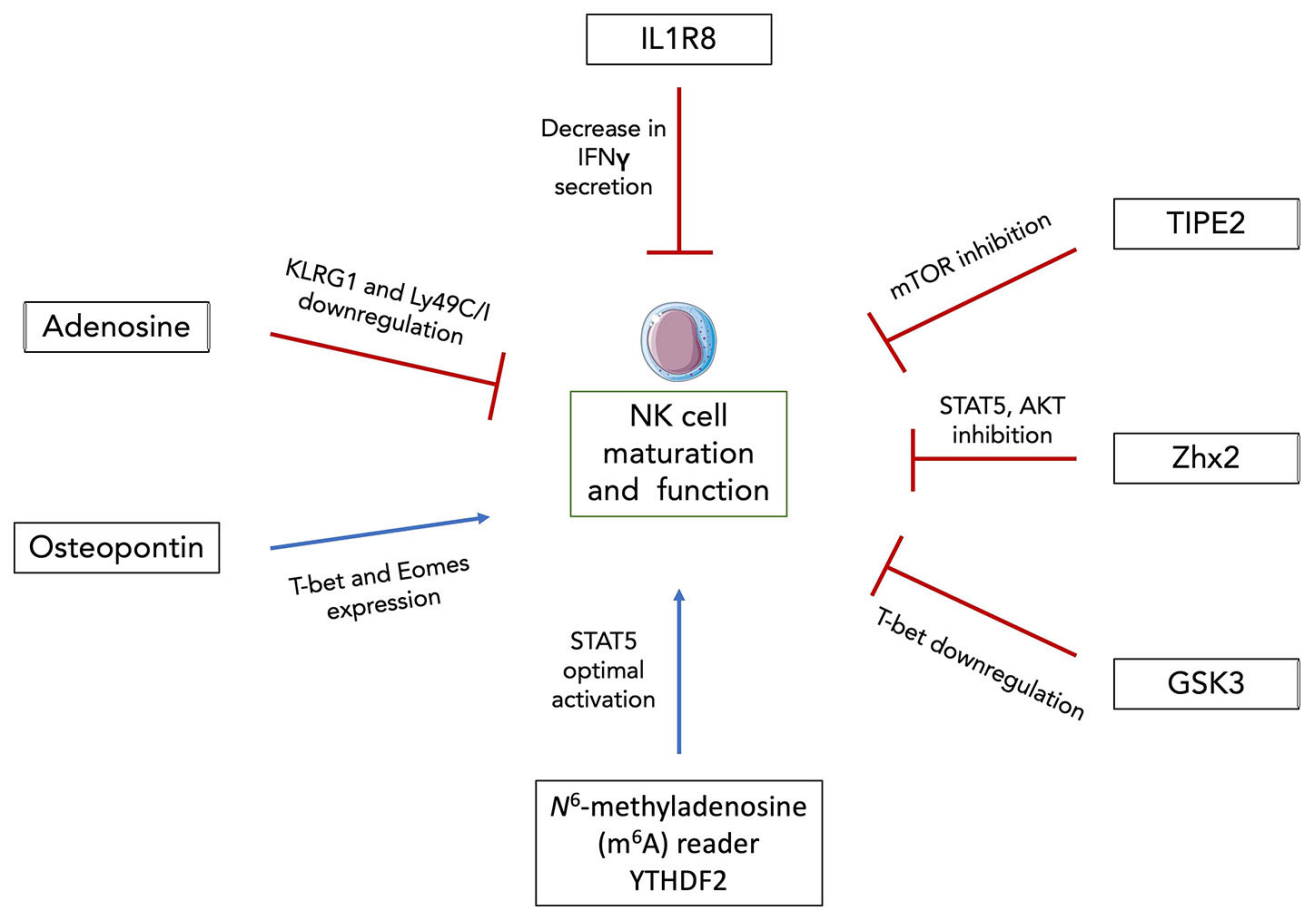
Newly discovered factors modulating NK cell maturation and function. Intracellular osteopontin (iOPN) and YTHDF2 were shown to positively regulate NK cell maturation and functional response. iOPN deficiency resulted in the downregulation of T-bet and Eomes expression as well as in the impairment of NK cell cytolytic activity and cytokine secretion. YTHDF2 promotes NK cell maturation, proliferation, survival and effector functions through a STAT5-YTHDF2 positive feedback loop. On the other hand, adenosine signaling, IL1R8, TIPE2, Zhx2 and GSK3 were all shown to negatively regulate NK cell maturation and anti-tumor activity through downregulation of maturation markers and/or transcription factors, decreased IFNγ secretion and inhibition of diverse NK cell activating signaling pathways. Thus, these factors constitute promising targets for NK cell-based immunotherapies.

Adenosine is an immunosuppressive metabolite generated in response to hypoxia or extracellular stress (91). It signals through 4 different receptors (A1, A2A, A2B and A3) and it was shown that signaling through A2AR prevents the infiltration of lymphocytes CD8^+^ T cells as well as NK cells (92, 93). *In vitro* culture of NK cells with adenosine and adenosine analogues leads to decreased cytotoxicity and cytokine production (94). However, Young et al. went further by studying the impact of A2AR signaling on NK cell maturation and anti-tumor response *in vivo* (95). They showed that NK cell-specific deletion of the A2AR signaling led to an accumulation of terminally mature CD11b^+^ mNK cells in the BM and accordingly decreased numbers of DP NK cells. The expression of maturation markers such as KLRG1 and Ly49C/I were also higher in CD11b^+^ mNK cells from Ncr1^iCre^ x A2AR^flox^ mice compared to littermate controls (**Figure 2**). They performed BM reconstitution with NK derived from Ncr1^iCre^ x R26^YFP^ and Ncr1^iCre^ x A2AR^flox^ mice at a 1:1 ratio into irradiated CD45.1^+^ recipients. After 14 weeks, A2AR-deficient NK cells displayed significantly greater reconstitution in central and peripheral organs (95). They also displayed a more mature KLRG1^+^ CD27^-^ CD11b^+^ phenotype than their counterparts from Ncr1^iCre^ R26^YFP^ mice indicating a cell-intrinsic effect of adenosine signaling. Subcutaneous injections of SM1WT1 melanoma cells into Ncr1^iCre^xA2AR^flox^ mice revealed reduced tumor growth and enhanced NK cell infiltration compared to littermate controls. The proportion of CD11b^+^ mNK cells was increased in the subcutaneous tumor while that of DP NK cells was decreased. Similar results were obtained in a model of MCA-induced fibrosarcoma. Lastly, Young et al. cultured human NK cells with IL-2 and A2AR inhibitor for 6 days which resulted in a decreased proportion of CD56^bright^ NK cells (95). Since CD56^brigh^ NK cells are thought to differentiate into CD56^dim^ NK cells, these results suggest that adenosine could negatively regulates NK cell maturation and anti-tumor activity both in mice and humans.

Osteopontin (OPN) previously known as Secreted phosphoprotein (Spp1) is a phosphorylated glycoprotein involved in homeostatic (biomineralization, wound healing, cell viability…) as well as pathological situations (cancer, inflammation, metabolic diseases…) (96). Two isoforms of OPN exist, one that is secreted (sOPN) and the other that remains intracellular (iOPN) as it lacks the N-terminal signal sequence (97). OPN is expressed in a large variety of tissues and by a substantial amount of cell types. It was shown that OPN skews the balance of BM lymphoid and myeloid population sizes (98). Notably, iOPN expression in myeloid progenitors tends to decrease their numbers and consequently that of mature myeloid cells by promoting apoptosis of the progenitors. On the other hand, BM-derived sOPN increases the differentiated lymphoid population size. Another study showed that *Spp1* transcript expression starts in the first mature stage of BM NK cells and is increased along their development with a peak of expression at the DP stage (99). Using OPN-deficient (OPN KO) mice and iOPN-knock-in (iOPN KI) mice expressing only the intracellular isoform (but not the secreted one), they showed that iOPN participated to NK cell maturation. BM reconstitution experiments demonstrated an intrinsic NK cell effect. Sublethally irradiated *Rag2^−/−^γ_C_* ^−/−^ hosts were injected with a mixture of NK cells from OPN KO and iOPN KI in CD45.1^+^ congenic mice. After 12 weeks, iOPN-sufficient NK cells displayed a twofold greater reconstitution than their OPN-deficient counterparts. Moreover, iOPN deficiency leads to the downregulation of T-bet and Eomes (**Figure 2**) as well as to an impairment of NK cell function with decreased cytolytic activity and cytokine secretion (99). However, these studies were performed mainly under homeostatic conditions. Given that OPN production is usually dysregulated in inflammatory contexts (100–103), it could be interesting to investigate the impact that OPN might have on NK cell maturation and functional response in such cases.

### Peripheral regulation of NK cell functional maturation

In the periphery, the *N* ^6^-methyladenosine (m^6^A) reader YTHDF2 was shown to positively regulate NK cell maturation, proliferation, survival and effector functions *via* a STAT5-YTHDF2 positive feedback loop (104). m^6^A is a common posttranscriptional modification on mRNA and is involved in a variety of biological contexts (105). YTHDF2 binds to methylated RNA and can promote translation as well as regulation of its stability (106). Using Ncr1^iCre^xYthdf2^flox^ mice, Ma et al. demonstrated that YTHDF2 is highly expressed in NK cells and specific deletion of *Ythdf2* in NK cells leads to an impairment of their antitumor and antiviral activities. Intravenous injection of B16F10 tumor cells in Ncr1^iCre^xYthdf2^flox^ mice revealed a significant reduction in infiltrating YTHDF2-deficient NK cells compared to their WT counterparts accompanied by a decreased expression of IFNγ, Granzyme B and perforin. Next, infections with MCMV showed that YTHDF2-deficient mice displayed decreased numbers of Ly49H^+^/Ly49D^+^ NK cells (104). This resulted in heavier viral loads suggesting that YTHDF2 is involved in Ly49-mediated NK cell response against MCMV. Besides its impact on NK cell functions, YTHDF2 is upregulated along the maturation process of NK cells with terminally differentiated CD27^-^CD11b^+^ mNK cells expressing it at the highest level. At steady state in periphery, Ncr1^iCre^xYthdf2^flox^ mice display an increase in DP NK cells and subsequent drop in CD27^-^CD11b^+^ mNK cell numbers compared to their littermate controls. Consistent with these findings, expression levels of KLRG1 on YTHDF2-deficient CD11b^+^ mNK cells were significantly lower. BM reconstitution experiments revealed a reduction in the proportion of CD11b^+^ mNK cells derived from BM YTHDF2-deficient cells with decreased Eomes expression at both mRNA and protein levels. Thus, YTHDF2 potentially promotes NK cell terminal maturation by regulating Eomes. Lastly, using Ncr1^iCre^xStat5^flox^ mice, they demonstrated that YTHDF2 is downstream of IL-15/STAT5 signaling while STAT5 phosphorylation is partially inhibited in Ncr1^iCre^xYthdf2^flox^ mice suggesting that YTHDF2 is required for optimum STAT5 activation (**Figure 2**) (104). Thus, this study revealed the existence of a new positive NK cell maturation regulator although it could be of interest to translate these results to human NK cells.

However, most studies have highlighted negative regulators of NK cell functional maturation and are proposing them as targets for enhancing NK cell-mediated tumor surveillance. IL-1R8 is part of the IL-1 receptor family and is known to negatively control immune responses and T cell functions (107). Human NK cells express high levels of IL-1R8 at the mRNA and protein levels and the latter increase along the developmental pathway. In mice, *Il1R8* transcript expression rise particularly during the maturation process, from CD27^+^CD11b^-^ to CD27^-^CD11b^+^ mNK cell stages (108). Using IL-1R8 KO mice, Molgora et al. found increased numbers of circulating, liver and splenic NK cells compared to WT mice (108). In the BM, the frequency of terminally differentiated KLRG1^+^ mNK cells were higher suggesting greater *in situ* maturation even if homing of peripheral activated NK cells could not be excluded. The frequency of NK progenitors on the other hand remained unchanged indicating that IL-1R8 is not required for early development of NK cells. Using different models of cancer as well as MCMV infection, they were able to demonstrate that IL-1R8 deficiency in NK cells was associated with enhanced antitumor and antiviral activities. Expression of activating receptors (NKG2D, CD226, Ly49H) were increased in peripheral blood NK cells from *Il1R8^-/-^* mice. IFNγ and Granzyme B secretion were more sustained in from *Il1R8^-/-^* splenic NK cells upon *ex vivo* stimulation with IL-18. Culture of healthy donor-derived human NK cells with a combination of IL-12 and IL-18 revealed an inverse correlation between IL-1R8 expression and levels of secreted IFNγ (**Figure 2**). Partial silencing of *Il1R8* with small interfering RNA led to an increased IFNγ production in IL-1R8-deficient human NK cells (108). Thus, IL-1R8 is a negative regulator of NK cell maturation and effector functions in mice and humans although the impact of IL-1R8 on human NK cell development in the BM was not assessed.

In another study by Bi et al., TNF-α–induced protein-8 like-2 (TIPE2), a known apoptosis regulator, is shown to suppress NK cell maturation and antitumor activity (109). TIPE2 expression correlates with mouse and human NK cell maturation process and NK-specific TIPE2 deficiency in Ncr1^iCre^xTipe2^flox^ mice resulted in increased CD27^-^CD11b^+^ NK cell numbers in central and peripheral organs and accompanied by an increase in KLRG1 expression on splenic NK cells. BM chimera experiments indicated that TIPE2 intrinsically inhibits NK cell maturation. In *in vitro* cytotoxic assays, TIPE2-overexpressing human NK cell lines showed decreased cytolytic activity while PMA/ionomycin-stimulated TIPE2-deficient murine NK cells displayed higher levels of CD69, IFNγ and CD107a demonstrating that TIPE2 suppresses NK cell activation and cytolytic activity in both mice and humans (109). Moreover, NK cells from Ncr1^iCre^xTipe2^flox^ mice proliferate more in response to IL-15 suggesting an enhanced IL-15 signaling in the absence of TIPE2. Mechanistically, this can be explained by the fact that TIPE2 inhibits IL-15 mediated mTOR activation (**Figure 2**) as *in vitro* culture of NK cells in presence of mTOR inhibitor rapamycin abrogates the enhanced proliferation and activation of TIPE2-deficient NK cells as well as the inhibitory effect of TIPE2 overexpression. Lastly, intravenous injections of diverse tumor cell types in Ncr1^iCre^xTipe2^flox^ mice show decreased tumor growth and higher numbers of NK cells suggesting that TIPE2 inhibits NK cell antitumor immunity.

Using similar techniques and approaches, Tan et al. showed that transcription factor Zinc fingers and homeoboxes 2 (Zhx2) negatively modulates NK cell maturation and antitumor response (110). Indeed, Ncr1^iCre^xZhx2^flox^ mice display an accumulation of CD27^-^CD11b^+^ KLRG1^+^ NK cells in the BM, spleen, liver and blood in comparison to Ncr1^iCre^xZhx2^WT^ mice indicating a more mature phenotype. Mixed BM chimera experiments were performed by transfer of CD45.2^+^ Zhx2-deficient cells and CD45.1^+^ Zhx2-sufficient cells at 1:1 ratio in irradiated CD45.1^+^CD45.2^+^ hosts. Splenic NK cell chimerism was assessed 8 weeks after injection and revealed increased number of NK cells derived from Zhx2-deficient cells indicating that the role of Zhx2 is cell intrinsic. Effector functions (IFNγ, TNFα, granzyme B, CD107a) and cytolytic activity are enhanced in Ncr1^iCre^xZhx2^flox^ mice compared to their WT counterparts (110). Gene Set Enrichment Analysis using RNA-seq data of splenic NK cells from Ncr1^iCre^xZhx2^flox^ and their littermate control mice revealed an enrichment for IL-15 signaling-associated genes in Zhx2-deficient NK cells. More specifically, they showed that IL-15 stimulated NK cells from Ncr1^iCre^xZhx2^flox^ mice display increased levels of phosphorylated STAT5 and AKT suggesting that Zhx2 regulates NK cell response to IL-15 through the activation of these downstream signaling pathways (**Figure 2**). Moreover, transcriptomic analyses led to the identification of *Zeb*2 as direct target of Zhx2. Transcription factor Zeb2 was reported to be essential for terminal maturation of NK cells as well as survival of CD27^-^CD11b^+^ mNK cells (111) thus supporting the idea that Zhx2 restricts NK cell maturation by repressing Zeb2 expression. Lastly, using an orthotopic model of HCC, Tan et al. showed that Ncr1^iCre^xZhx2^flox^ mice displayed higher levels of tumor infiltrating mNK cells with enhanced CD107a expression compared to control mice suggesting a better control of tumor growth by Zhx2-deficient NK cells.

In a study from 2017, Glycogen synthase kinase (GSK) 3, a constitutively active serine-threonine kinase, was reported to restrict NK cell maturation and antitumor activity (112). GSK3 can impact gene expression either by directly targeting transcription factor or by phosphorylating histones (113). Cochocki et al. isolated NK cells from peripheral blood of healthy MCMV seropositive donors and cultured them for 7 days with IL-15, with or without GSK3 selective inhibitor CHIR99021. Overall expansion of cells was similar despite increased proportion of CD57^+^NKG2C^+^ NK cells in the CHIR99021 condition compared to the control. The glycan carbohydrate CD57 molecule is expressed at the surface of terminally differentiated NK cells and CD57^+^NKG2C^+^ NK cells are considered as mouse Ly49H^+^ NK cell counterparts and referred to “adaptive” NK cells (114). The upregulation of CD57 was accompanied by that of *Tbx21* (**Figure 2**) and its two targets *Zeb2* and *Prdm1* both at the mRNA and protein levels (112) demonstrating an increased maturation of *ex vivo* expanded NK cells when GSK3 is inhibited. *Eomes* expression on the other hand was not impacted. In addition, cultures of NK cells with CHIR99021 displayed higher levels of IFNγ and TNFα as well as enhanced cytolytic activity towards several solid tumor cell lines. Lastly, Cochocki et al. injected luciferase-expressing SKOV-3 tumor cells in NSG mice before transferring NK cells primed overnight with IL-15 +/- CHIR99021. Tumor growth was followed over the course of 40 days *via* imaging of luciferase-positive cells. Mice that received IL-15 after priming of NK cells with CHIR99021, displayed better tumor control demonstrating that inhibition of GSK3 promotes NK cell antitumor activity after adoptive transfer. Although not discussed in the study, it is interesting to note that GSK3 is inhibited by AKT which is itself downstream of the IL-15/PI (3)K pathway (115). This further supports the fact that GSK3 inhibition contributes to enhancing IL-15 signaling and consequently NK cell functional maturation.

Altogether, these studies have uncovered new immune checkpoints in NK cell maturation and antitumor response in both mice and humans offering promising targets for NK cell-based immunotherapies.

## Impact of inflammation and therapy on NK cell development and maturation

Hematopoiesis is a tightly regulated process. However, the latter can be affected in a variety of inflammatory contexts. In the case of NK cells, due to their importance in diseases such as cancer and viral infections, it remains essential to uncover the impact the inflammation itself could have on their development, maturation and effector functions.

### Obesity

**Figure 3:**
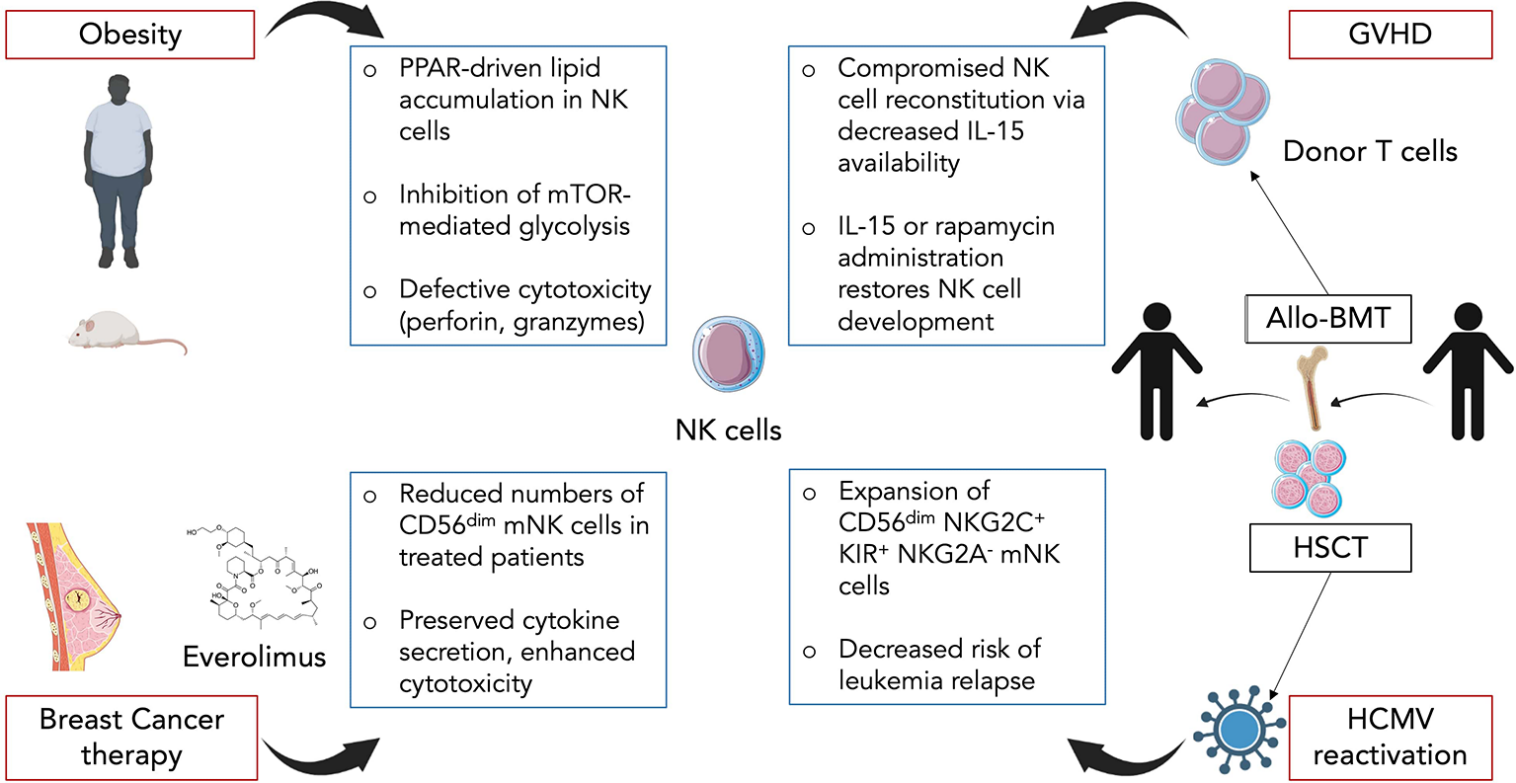
Impact of diverse inflammatory contexts on NK cell maturation and functional response. In obesity, circulating NK cells accumulate lipid droplets through a PPAR-driven mechanism. This directly promotes an inhibition of mTOR-mediated glycolysis as well as decreased cytotoxic capacity. In breast cancer therapy, mTOR inhibitor everolimus is commonly used and induces changes in NK cells. Treated patients display reduced numbers of CD56^dim^ mNK cells but the remaining cells exhibit preserved cytokine secretion along with enhanced target cell killing. In graft-versus-host-disease (GVHD) following allogeneic BM transplantation (Allo-BMT), donor T cells compete with NK cells for IL-15 resulting in a compromised NK cell reconstitution. Administration of exogenous IL-15 or rapamycin helps restore NK cell development. In hematopoietic stem cell transplant (HSCT) recipients, human cytomegalovirus (HCMV) reactivation promotes expansion of mature CD56^dim^NKG2C^+^KIR^+^NKG2A^-^ NK cells. HCMV reactivation also correlates with decreased risk of leukemia relapse which could be linked to the functional status of NK cells.

Obesity rates have skyrocketed worldwide for the past decades, especially in western countries. The unbalanced diets are now responsible for a high prevalence in the adult population, almost reaching 40% in the United States of America. Obesity predisposes individuals to metabolic disorders such as type 2 diabetes but also to infectious diseases (116). This can be accounted for by the fact that being overweight can compromises one’s immunity and dysregulates the immune system (117). Indeed, NK cells are known to be dysregulated in obesity both in mice and humans (118, 119). More specifically, Michelet et al. showed how obesity is responsible for a peroxisome proliferator-activated receptor (PPAR)-driven lipid accumulation in NK cells which inhibits mTOR-mediated glycolysis and leads to defective cytotoxicity (120) (**Figure 3**). Using mouse models of diet-induced obesity, they performed transcriptional analysis on NK cells which unveiled the upregulation of lipid metabolism-related genes and the downregulation of genes involved in the cytotoxic response and the mTOR signaling pathway. The same pathways were altered in NK cells from obese humans. *In vitro* culture of human NK cells with free fatty acids (FFAs) revealed that the more NK cells accumulated lipid droplets the less they expressed perforin and granzyme B (**Figure 3**). This demonstrates a direct causality between lipid uptake and decreased cytolytic activity in NK cells. In accordance with these findings, lipid-treated NK cells failed to control tumor growth *in vivo* in a B16F10 melanoma model (120). In addition, they showed that NK cells isolated from obese patients displayed a significantly diminished mTORC1 activation after cytokine stimulation as revealed by lower levels of the phosphorylated S6 ribosomal protein, a target of mTORC1 (120). Although not discussed in the study, it is interesting to note that circulating leptin levels are increased during obesity alongside with the development of a leptin resistance. Leptin is a peptide hormone produced by adipocytes which regulates body weight *via* a negative feedback loop between the hypothalamus and the adipose tissue (121) and was shown to activate the PI3K/Akt/mTOR pathway (122). Since this activation is sensitive to rapamycin, leptin most likely activates mTOR *via* the mTORC1. Thus, we can hypothesize that impaired leptin signaling in obesity and more specifically in NK cells might contribute to the dysregulation of the mTOR pathway. Moreover, given that mTOR is essential for IL-15 signaling and consequently for NK cell development and activation (123), it can be assumed that obesity not only affects NK cell cytotoxic functions and antitumor activity but may also impact NK cell development and maturation. Examining NK cell development and maturation as well as measuring leptin levels in the BM of obese mice and patients would help answer that pending question.

### Cancer therapy

Cancer is the second cause of mortality worldwide accounting for almost 10 million deaths in 2018 according to the World Health Organization. In women, breast cancer (BC) is the leading cancer in terms of incidence and each year millions of BCs are diagnosed (124). Besson et al. studied the impact that mTORC1 inhibition in metastatic BC has on NK cell maturation, numbers and functions (125). Cancer cells usually display an enhanced activation of the PI3K/AKT/mTOR pathway to sustain their metabolic needs. Moreover, mTOR expression is associated with poor prognostic in BC patients (126). Therefore, everolimus an analog of rapamycin is frequently used in resistant forms of BC. Given that mTOR is required for NK cell development and activation (71, 123), everolimus might have a detrimental effect on NK cell antitumoral response. To answer that question, they analysed blood samples from metastatic BC patients before beginning the treatment (baseline), 1 and 3 months after (M1 and M3). By measuring the levels of phosphorylation of ribosomal protein S6 and AKT, respectively downstream of mTORC1 and mTORC2, they were able to conclude that everolimus leads to a specific inhibition of mTORC1 and does not affect mTORC2. Circulating NK cell numbers were gradually decreased from baseline to M3, with a restricted reduction of CD56^dim^ NK cells while numbers of CD56^bright^ NK cells remained unchanged, suggesting a possible default of maturation (**Figure 3**). Consistent with this hypothesis, expression levels of maturation-associated markers CD57, KLRG1, CD16, 2B4 and CD161 were downregulated. Interestingly, NK cells isolated at M3 displayed preserved cytokine secretion ability and even an increased target cell killing in a cytotoxicity array (125) (**Figure 3**). These results contrast with those obtained in mouse models where mTOR activity was positively linked to NK cell cytokine secretion and cytotoxic response (120, 127). The discrepancies could be explained by differences between the two species, an indirect effect of everolimus or from the pathological context of BC. Thus, even though inhibition of mTORC1 leads to a defect in mature NK cells, it could be beneficial to cytolytic functions of the remaining cells, a possible notion which challenges previous findings in other models.

### GVHD

Allogeneic BM transplantation (allo-BMT) is a curative therapy used to replace stem cells that were destroyed during irradiation or chemotherapy. It consists in a donor giving BM cells to a recipient (128). However, allo-BMT is associated with several complications such as opportunistic infections or graft-versus-host-disease (GVHD). GVHD is a common condition after allo-BMT, occurring in 50% to 70% of patients, and is caused by donated BM cells recognizing the recipient’s body cells as foreign and thus attacking them (129, 130). It is mostly mediated by donor T cells and pro-inflammatory cytokines. Given that NK cells harbor both antiviral and antitumor activities, they are highly effective for fighting leukemia and/or CMV reactivation in transplanted patients. Yet, Bunting et al. showed how donor T cells in GVHD prevent proper NK cell reconstitution and thus compromise NK cell immunity (131). Using B6-background strains of mice, they created a BM transplant model with GVHD targeting both MHC class I and II. A significant impaired reconstitution of NK cells in the BM and an even stronger defect in NK cells in periphery (spleen, liver, lung, lymph nodes) were observed in mice with GVHD (allogeneic transplant) compared to non-GVHD mice (syngeneic transplant) (**Figure 3**). Increased apoptosis in the early phase of GVHD partially explained the diminished splenic NK pool as revealed by Annexin V staining experiments. In the BM, the decrease in NK cell numbers started at the CD27^+^ NK stage suggesting a default in the maturation process. In order to determine whether donor T cells were responsible for NK cell decreased numbers, Bunting et al. used T cell-depleted grafts and compared them to T cell-repleted ones. In T cell-depleted grafts, higher numbers of NK cells were observed regardless of the presence of GVHD suggesting a competition between donor T cells and NK cells for expansion (131). IL-15 is essential for NK cell maturation, survival and proliferation but is also required for CD8^+^ T cell homeostasis. Indeed, levels of IL-15/IL-15Rα complex were significantly lower in T cell-repleted grafts compared to T cell-depleted ones. Transplantation with BM cells with IL-15Rα^-/-^ T cells resulted in an enhanced NK cell reconstitution. Administration of exogenous IL-15/IL-15Rα complex in GVHD-affected mice led to increased NK cell numbers compared to non-treated mice (**Figure 3**). Altogether, these results point towards a competition between T cells and NK cells for IL-15 consumption thereby limiting the expansion of the latter. Interestingly, use of the mTOR inhibitor rapamycin led to a similar result as IL-15/IL-15Rα injections although mTOR was shown to be essential for IL-15 signaling-mediated NK cell development and activation (71, 123). This is likely due to the fact that rapamycin primarily restricts donor T cell activation which counterbalances its own negative effects on NK cells. Lastly, the defect in NK cells induced by GVHD resulted in the failure of NK cells to control CMV infection as well as tumor cells in an acute myeloid leukemia model. Thus, this study highlights the effect of GVHD on NK cell maturation and functional response and proposes solutions with IL-15 and rapamycin administration.

### Viral infection

Human CMV (HCMV) is a very common herpesvirus, infecting between 50% and 100% of individuals depending on the geographical location and socioeconomic factors (132). Most people remain infected for life with occasional reactivations especially in immunosuppressed or immunodeficient patients (133). Hematopoietic stem cell transplantation (HSCT) recipients are thus particularly at risk. NK cells are usually the first lymphocytes to reconstitute after transplantation and play a crucial role in the control of the HCMV. In murine CMV infection, a population of Ly49H^+^ NK cells was shown to expand and display “memory-like” properties (134). In humans, expansion of NK cells expressing high levels of the activating receptor CD94/NKG2C specific for non-classical HLA-E was observed in patients infected with HCMV (135, 136). These expanded NK cells exhibit a mature CD56^dim^NKG2C^+^KIR^+^NKG2A^-^phenotype (**Figure 3**). Thus, several studies took an interest in how HCMV reactivation could impact NK cell reconstitution in HSCT recipients (137, 138). Using patients having received umbilical cord blood transplantations, they analysed NK cell development up to 12 months after transplant. NK cells were the first lymphocyte population to be detected in peripheral blood however NK cells from patients with HCMV reactivation expressed a mature phenotype while other patients harbored a majority of immature NK cells (137). These persisting NKG2C^+^ NK cells preferentially upregulated CD57 and expressed higher levels of IFNγ, IL-15Rα and T-bet mRNA transcripts compared to patients without HCMV reactivation (138). Lastly, Elmaagacli et al. reported that HCMV reactivation in HSCT recipients correlated with a decreased risk of leukemia relapse (**Figure 3**) (139) supporting the idea that HCMV reactivation might actually be beneficial for shaping full mature and functional NK cells protecting the host.

**Figure 4:**
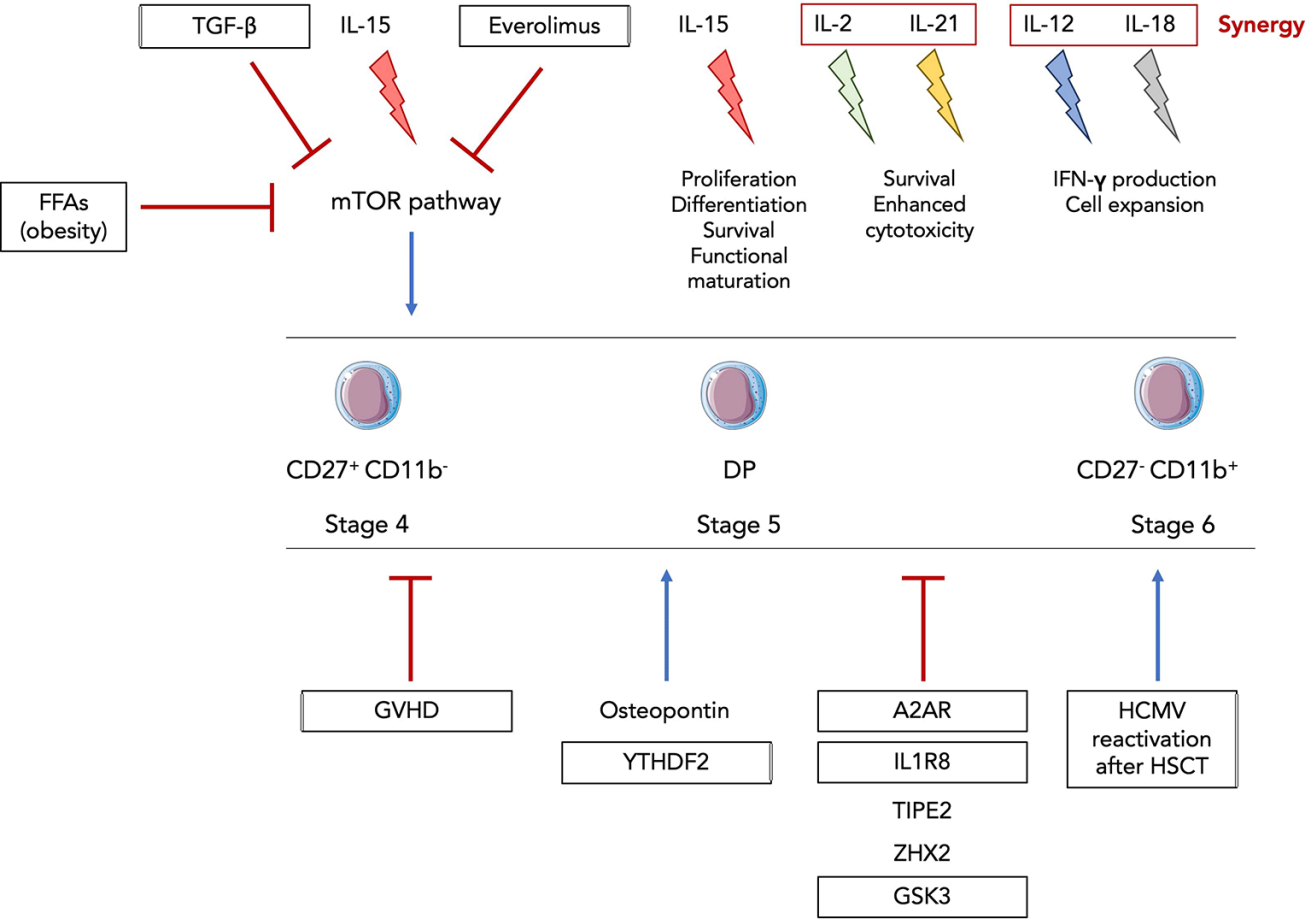
Inflammatory-driven regulation of NK cell functional maturation. The family of common γc cytokines is known to be involved in NK cell development and functional activation. IL-15 is the main cytokine driving proliferation, differentiation, survival and functional maturation and can act on all stages of murine and human CD122^+^ NK progenitors and precursors. IL-2 and IL-21 can synergize and promote survival of mouse and human NK cells as well as enhance their cytotoxic activity. IL-12 and IL-18 also synergize to activate NK cells and amplify their expansion and production of IFNγ (140). Everolimus, free fatty acids (FFAs) in obsesity and TGF-β were shown to inhibit the IL-15-mediated activation of the mTOR pathway. GVHD, A2AR, IL1R8, TIPE2, ZHX2 and GSK3 were discovered as newly factors and pathological context negatively regulating NK cell functional maturation while osteopontin, YTHDF2 and HCMV reactivation after HSCT promote it. Framed factors and inflammatory contexts were validated in human NK cells.

Altogether, these studies constitute novel approaches in the study field on NK cell maturation (**Figure 4**). More studies are necessary to uncover how inflammatory contexts dysregulate NK cell development, especially in the BM, and the consequences this has on their functional response in pathology.

## Conclusion

Understanding what mechanisms are at play in the regulation of NK cell development and maturation will help design new therapeutic approaches using these cells. Yet, too few studies have focused on the impact inflammation itself has on NK cell development and maturation. In a Lewis lung cancer model, tumor progression was positively correlated with infiltration of CD27^-^CD11b^-^ NK cells suggesting that the tumor microenvironment locally regulates NK cell maturation process (141). In other primary and metastatic cancer settings, TGF-β was shown to be upregulated and to drive conversion of effector NK cells into ILC1-like cells (142) highlighting their potential of inflammation-mediated plasticity. Another crucial feature of NK cells is their ability to mobilize both innate and antigen-specific immune responses. It would be interesting to determine whether “memory-like” NK cells undergo different steps of maturation. Thus, the study of NK cell development and maturation under inflammation remains open and requires further investigation.

## Author contributions

EB designed and prepared the manuscript and the figures. RG gave guidance on the outline and revised the manuscript. All authors contributed to the article and approved the submitted version.
